# Isolation and Characterization of *Klebsiella* Phages for Phage Therapy

**DOI:** 10.1089/phage.2020.0046

**Published:** 2021-03-17

**Authors:** Eleanor M. Townsend, Lucy Kelly, Lucy Gannon, George Muscatt, Rhys Dunstan, Slawomir Michniewski, Hari Sapkota, Saija J. Kiljunen, Anna Kolsi, Mikael Skurnik, Trevor Lithgow, Andrew D. Millard, Eleanor Jameson

**Affiliations:** ^1^ Department of Microbiology and Virology, School of Life Sciences, Gibbet Hill Campus, The University of Warwick, Coventry, United Kingdom.; ^2^ Department of Genetics, University of Leicester, Leicester, United Kingdom.; ^3^ Infection and Immunity Program, Department of Microbiology, Biomedicine Discovery Institute, Monash University, Melbourne, Australia.; ^4^ Department of Neuroscience, Psychology and Behaviour, University of Leicester, Leicester, United Kingdom.; ^5^ Department of Bacteriology and Immunology, Human Microbiome Research Program, Faculty of Medicine, University of Helsinki, Helsinki, Finland.; ^6^ Division of Clinical Microbiology, Helsinki University Hospital, HUSLAB, Helsinki, Finland.

**Keywords:** Klebsiella, bacteriophage, phage, phage therapy, antimicrobial resistance, antibiotics, nosocomial infection, characterization, virulence

## Abstract

***Introduction:*** *Klebsiella* is a clinically important pathogen causing a variety of antimicrobial resistant infections in both community and nosocomial settings, particularly pneumonia, urinary tract infection, and sepsis. Bacteriophage (phage) therapy is being considered a primary option for the treatment of drug-resistant infections of these types.

***Methods:*** We report the successful isolation and characterization of 30 novel, genetically diverse *Klebsiella* phages.

***Results:*** The isolated phages span six different phage families and nine genera, representing both lysogenic and lytic lifestyles. Individual *Klebsiella* phage isolates infected up to 11 of the 18 *Klebsiella* capsule types tested, and all 18 capsule-types were infected by at least one of the phages.

***Conclusions:*** Of the *Klebsiella*-infecting phages presented in this study, the lytic phages are most suitable for phage therapy, based on their broad host range, high virulence, short lysis period and given that they encode no known toxin or antimicrobial resistance genes. Phage isolates belonging to the *Sugarlandvirus* and *Slopekvirus* genera were deemed most suitable for phage therapy based on our characterization. Importantly, when applied alone, none of the characterized phages were able to suppress the growth of *Klebsiella* for more than 12 h, likely due to the inherent ease of *Klebsiella* to generate spontaneous phage-resistant mutants. This indicates that for successful phage therapy, a cocktail of multiple phages would be necessary to treat *Klebsiella* infections.

## Background

The *Klebsiella* genus causes pneumonia, urinary tract infection, and sepsis, particularly in vulnerable populations, often causing secondary infections in ventilated or catheterized patients, in both the community and nosocomial settings.^1–4^ In addition, the subclinical carriage of *Klebsiella* is linked to cardiovascular^5,6^ and inflammatory bowel disease.^7^ *Klebsiella pneumoniae* is the most problematic species giving rise to hypervirulent clones with extended virulence factors.^8–11^

Antimicrobial resistance (AMR) represents a threat to global health and security, fueled by our intensive use of antibiotics in medicine and agriculture. *Klebsiella* readily gains and transfers AMR genes, particularly in health care settings, making it a World Health Organization priority pathogen.^12,13^ The prevalence of multi-drug resistant (MDR) *Klebsiella* has increased exponentially to most available antimicrobial drugs, and cases of pan-resistant *Klebsiella* are now common around the world.^14–17^ MDR *Klebsiella* infections pose an increased risk of mortality,^18,19^ are difficult to treat^20,21^ and outbreaks are economically costly.^19^ Bacteriophages (phages)^22^ offer one potential alternative treatment.

Phage therapy is a potential weapon against MDR bacterial infections.^23,24^ Phage therapy depends on preparedness, particularly in having a real or virtual “biobank” of characterized phages against common AMR bacteria. Phage characterization is essential to provide effective, timely treatment and mitigate side effects.^25^

In this article, we use genomic and imaging technologies to characterize novel phages isolated against *Klebsiella* spp. Phages were isolated from rivers, ponds, estuaries, canals, slurry, and sewage. Their characterization focused on their infection cycle, host range, and gene content. We present phages with siphovirus, myovirus, podovirus, and inovirus morphologies, spanning six phage families and nine genera, of which the majority have lytic lifestyles. A number of these phages have potential use in phage therapy.

## Materials and Methods

### Bacterial strains and culture conditions

The *Klebsiella* strains used in this work are listed in Table 1. All culturing in liquid medium was performed with shaking (150 rpm) at 37°C. All culturing was carried out in Lysogeny broth (LB), with the addition of 5 mM CaCl_2_ and MgCl_2_. The *Klebsiella* were originally isolated from clinical and environmental samples (Table 1). They represented six species: *K. pneumoniae*, *Klebsiella oxytoca*, *Klebsiella quasipneumoniae*, *Klebsiella variicola*, *Klebsiella michagenesis*, and *Klebsiella aerogenes*.

**Table 1.** Details of *Klebsiella* Species and Strains Used in This Study

Species	Strain	Capsule (K) and LPS antigen (O) locus	Isolation host?	Origin	Isolation source
*Klebsiella aerogenes*	30053	—	✓	DSMZ Culture Collection	Sputum
*Klebsiella michiganensis*	25444	O1v1	×	DSMZ Culture Collection	Toothbrush holder
*Klebsiella oxytoca*	170748	O1v1	×	Clinical Isolate	Catheter specimen urine
	5175	KL29	×	DSMZ Culture Collection	Pharyngeal tonsil
	25736	KL74	✓	DSMZ Culture Collection	Case of pneumonia
	170821	OL104	✓	Clinical Isolate	Urine
	171266	OL104	×	Clinical Isolate	Urostomy urine
*Klebsiella pneumoniae*	170958	KL28	✓	Clinical Isolate	Urine
	171304	KL144	×	Clinical Isolate	Catheter specimen urine
	13440	KL38	✓	NCTC Culture Collection	Clinical
	13442	KL110	×	NCTC Culture Collection	Hospital, Italy
	30104	KL3	✓	DSMZ Culture Collection	Human blood
	13465	KL57	×	NCTC Culture Collection	Clinical
	170820	KL158	×	Clinical Isolate	Urine
	16358	KL4	×	DSMZ Culture Collection	Human, nose
	170723	KL2	✓	Clinical Isolate	Urine
	171167	KL2	×	Clinical Isolate	Urine
	13443	KL2	×	NCTC Culture Collection	Clinical
	13882	KL64	×	ATCC Culture Collection	Water
	13439	KL14	✓	NCTC Culture Collection	Outbreak strain
*Klebsiella variicola*	W12	KL14	×	Environmental Isolate	Soil
	15968	KL16	✓	DSMZ Culture Collection	Banana root
*Klebsiella quasipneumoniae*	28211	KL35	✓	DSMZ Culture Collection	Human blood
	700603	KL53	✓	ATCC Culture Collection	Urine

Capsule and LPS antigen locus types are given where applicable, alongside the origin of the strain and indication of use as an isolation host.

LPS, lipopolysaccheride.

No ethical approval was required for this work.

### *Klebsiella* capsule typing

Kaptive Web was used to determine the capsule (K) and LPS antigen (O) locus types of the 24 *Klebsiella* strains.

### Phage isolation

Phages were isolated from water samples from various sources, listed in Table 2. Water samples were filtered through 0.2 μm pore-size syringe filters to remove debris and bacteria. Phages were then isolated by enrichment: 2.5 mL of filtered water was added to 2.5 mL nutrient broth, containing 5 mM CaCl_2_ and 5 mM MgCl_2_, and inoculated with 50 μL of overnight-grown *Klebsiella*. This enrichment culture was then incubated overnight at 37°C and centrifuged, and the supernatant was filtered through a 0.2 μm pore-size filter to remove cells. This filtrate was serially diluted to 10^−11^ in LB and used in an overlay agar plaque assay.

**Table 2.** Phage Isolate Details

Phage name	Lab ID	Life style lytic (L) or temperate (T)	Source of isolation		Strain of isolation	Capsule (K) and LPS antigen (O) locus	Accession no.
Klebsiella phage vB_KppS-Eggy	49	T	Sewage—anoxic sludge	Spernal sewage works, United Kingdom	*Klebsiella pneumoniae* DSM 30104	KL3	PRJEB40146
Klebsiella phage vB_KppS-Pokey	50	T	Sewage—anoxic sludge	Spernal sewage works, United Kingdom	*K. pneumoniae* DSM 30104	KL3	PRJEB40147
Klebsiella phage vB_KppS-Raw	33	T	Sewage—raw	Spernal sewage works, United Kingdom	*K. pneumoniae* DSM 30104	KL3	PRJEB40132
Klebsiella phage vB_KppS-Ant	35	T	Sewage—anoxic sludge	Spernal sewage works, United Kingdom	*K. pneumoniae* DSM 30104	KL3	PRJEB40148
Klebsiella phage vB_KaS-Ahsoka	7	L	Slurry	Slurry tank, United Kingdom	*Klebsiella aerogenes* DSM 30053	—	PRJEB40160
Klebsiella phage vB_KaS-Gatomon	6	L	Marine canal	Grand canal, Venice, Italy	*K. aerogenes* DSM 30053	—	PRJEB40159
Klebsiella phage vB_KppS-Samwise	8	L	Slurry	Slurry tank, United Kingdom	*K. pneumoniae* DSM 30104	KL3	PRJEB40161
Klebsiella phage vB_KvM-Eowyn	4	L	Estuary	Jelitkowo, Poland	*Klebsiella variicola* DSM 15968	KL16	PRJEB40131
Klebsiella phage vB_KpP-Screen	46	L	Sewage—sieve	Spernal sewage works, United Kingdom	*K. pneumoniae* 170723	KL2	PRJEB40164
Klebsiella phage vB_KpP-Yoda	43	L	Sewage—storm tank	Spernal sewage works, United Kingdom	*K. pneumoniae* 170723	KL2	PRJEB40162
Klebsiella phage vB_KqP-Goliath	44	L	Sewage—raw	Spernal sewage works, United Kingdom	*Klebsiella quasipneumoniae* DSM 700603	KL53	PRJEB40163
Klebsiella phage vB_KaS-Benoit	1	L	Estuary	Jelitkowo, Poland	*K. aerogenes* DSM 30053	—	PRJEB39773
Klebsiella phage vB_KaS-Veronica	2	L	Marine canal	Grand canal, Venice, Italy	*K. aerogenes* DSM 30053	—	PRJEB40165
Klebsiella phage vB_KppS-Anoxic	52	L	Sewage—anoxic sludge	Spernal sewage works, United Kingdom	*K. pneumoniae* DSM 30104	KL3	PRJEB40170
Klebsiella phage vB_KppS-Jiji	27	L	Pond	Gneiwkowo, Poland	*K. pneumoniae* DSM 30104	KL3	PRJEB40168
Klebsiella phage vB_KppS-Ponyo	19	L	River	Gneiwkowo, Poland	*K. pneumoniae* DSM 30104	KL3	PRJEB40167
Klebsiella phage vB_KppS-Storm	34	L	Sewage—storm tank	Spernal sewage works, United Kingdom	*K. pneumoniae* DSM 30104	KL3	PRJEB40169
Klebsiella phage vB_KppS-Totoro	10	L	Estuary	Jelitkowo, Poland	*K. pneumoniae* DSM 30104	KL3	PRJEB40166
Klebsiella phage vB_KqM-Bilbo	38	L	Sewage—raw	Spernal sewage works, United Kingdom	*K. quasipneumoniae* DSM 28211	KL35	PRJEB40172
Klebsiella phage vB_KqM-LilBean	36	L	Sewage—raw	Spernal sewage works, United Kingdom	*K. quasipneumoniae* DSM 28211	KL35	PRJEB40171
Klebsiella phage vB_KqM-Westerburg	39	L	Sewage—raw	Spernal sewage works, United Kingdom	*K. quasipneumoniae* DSM 28211	KL35	PRJEB40173
Klebsiella phage vB_KoM-Liquor	61	L	Sewage—mixed liquor	Spernal sewage works, United Kingdom	*Klebsiella oxytoca* 170821	OL104	PRJEB40174
Klebsiella phage vB_KoM-MeTiny	68	L	Sewage—mixed liquor	Spernal sewage works, United Kingdom	*K. oxytoca* DSM 25736	KL74	PRJEB40179
Klebsiella phage vB_KoM-Pickle	12	L	Estuary	Jelitkowo, Poland	*K. oxytoca* DSM 25736	KL74	PRJEB40176
Klebsiella phage vB_KpM-KalD	67	L	Sewage—mixed liquor	Spernal sewage works, United Kingdom	*K. pneumoniae* DSM 13439	KL14	PRJEB40178
Klebsiella phage vB_KpM-Mild	65	L	Sewage—mixed liquor	Spernal sewage works, United Kingdom	*K. pneumoniae* DSM 13439	KL14	PRJEB40177
Klebsiella phage vB_KoM-Milk	62	L	Sewage—mixed liquor	Spernal sewage works, United Kingdom	*K. oxytoca* 170821	OL104	PRJEB40175
Klebsiella phage vB_KpM-SoFaint	70	L	Sewage—mixed liquor	Spernal sewage works, United Kingdom	*K. pneumoniae* DSM 13440	KL38	PRJEB40180
Klebsiella phage vB_KoM-Flushed	63	L	Sewage—mixed liquor	Spernal sewage works, United Kingdom	*K. pneumoniae* 170958	KL28	PRJEB40181
Klebsiella phage vB_KpM-Wobble	64	L	Sewage—mixed liquor	Spernal sewage works, United Kingdom	*K. pneumoniae* 170958	KL28	PRJEB40182

Lab ID refers to the laboratory identification number, source of isolation indicates where the water sample was collected for phage enrichment and isolation, and the strain of isolation indicates *Klebsiella* sp. strain on which three rounds of plaque assay isolation were performed. Accession numbers refer to the associated project accession numbers assigned by the ENA for each phage.

Briefly, 50 μL of each serial dilution was mixed with 0.5 mL of a single *Klebsiella* strain in the logarithmic growth phase (approximately OD_600nm_ 0.2) and incubated at room temperature for 5 min. To each serial dilution/cell mix, 2.5 mL of cooled, molten LB agar (0.4% weight/volume) was added and mixed by swirling. The molten agar mix was poured onto 1% LB agar plates. Overlay agar plates were allowed to set, then inverted, and incubated overnight at 37°C. From the plaque assay plates, single plaques were picked, mixed with 50 μL of LB, and filtered through a 0.22 μm pore-size spin filter (Costar Spin-X; Corning, United Kingdom). This filtrate underwent two further rounds of plaque assay to ensure that clonal phages were isolated. Phages were named by using the ICTV binomial system of viral nomenclature.^26^

### DNA extraction

DNA was extracted by using the phenol–chloroform method.^27^ Briefly, phage lysates were concentrated by using a protein column with a 30 kDa cutoff. Seven hundred fifty microliters of concentrated phage was treated with DNase I and Proteinase K, before phenol–chloroform, then overnight precipitation with ammonium acetate and ethanol at −20°C. The DNA was resuspended in 50 μL of molecular-grade water. For phage DNA with high protein contamination from the method described earlier, the Norgen Phage DNA Isolation Kit was used following the manufacturer's instructions. To assess the quantity and quality of DNA for sequencing, both a spectrophotometer-based method and Qubit were used.

### Genome sequencing

Sequencing was performed by MicrobesNG (Birmingham, United Kingdom); briefly, genomic DNA libraries were prepared by using Nextera XT Library Prep Kit (Illumina, San Diego) following the manufacturer's protocol with modifications: 2 ng of DNA were used as the input, and a polymerase chain reaction (PCR) elongation time of 1 min. DNA quantification and library preparations were carried out on a Hamilton Microlab STAR automated liquid handling system. Pooled libraries were quantified by using the Kapa Biosystems Library Quantification Kit for Illumina, on a Roche light cycler 96 quantitative PCR machine. Libraries were sequenced on the Illumina HiSeq by using a 250 bp paired end protocol.

### Bioinformatics

Contig and genome assembly was carried out by MicrobesNG; reads were trimmed with Trimmomatic 0.30, sliding window quality cutoff of Q15^28^; and SPAdes (v3.7) was used for *de novo* assembly.^29^ Genomes were annotated by using Prokka,^30^ with a custom database downloaded from Genbank as previously described.^31^ The capsule types of the *Klebsiella* strain genomes were predicted by using Kaptive.^32^

To determine phage taxonomy, phage isolate genomes were added to VIPtree^33^ and subject to BLASTn and tBLASTn against NCBI. The average nucleotide identity (ANI) of our phages was compared with the genomes identified from the methods described earlier by using orthoANI.^34^ Genomes with an ANI >95% were designated as the same species.^35^

For genus-level clustering, a shared protein network analysis was performed by using vConTACT2 (v0.9.13)^36^ with all phage genomes available (May 2020).^31^ The resulting network graph was visualized and annotated within Cytoscape (v3.8.0).^37^ Finally, sequence alignments were performed by using MAFFT (v7.271)^38^ on the DNA polymerase, large terminase subunit, and major capsid proteins of each phage isolate genus with the most closely related phage proteins. Phylogenetic trees were constructed with RaxML (v8.2.4)^39^ with 1000 bootstrap calculations by using the GAMMA model of heterogeneity and the maximum-likelihood method based on the JTT substitution matrix. Subsequent trees were visualized and annotated in R (v3.6.1) by using ggtree (v1.16.6)^40,41^ and phytools (v0.7-70).^42^

Depolymerases were predicted in the phage isolate's structural genes, through enzymatic domains or features common to characterized depolymerase proteins. Each prediction was analyzed by BLASTP (v2.10.0), Pfam HMMER (v3.3), and HHpred (v33.1) by using the default settings. Sequences from biochemically characterized depolymerase proteins that target *Klebsiella* spp. (Supplementary Table S1) or the putative depolymerases from our phage isolates (Supplementary Table S2) were used for analysis. Sequences were aligned with Muscle (v3.8.31)^43^ by using SeaView (v4).^44^

Phylogenetic tree construction was performed with MegaX^45^ with 500 bootstrap calculations by using the LG model. Tree topology searches were performed by using a combination of NNI and NJ/BioNJ. The tree was subsequently visualized and annotated by using iTOL(v4).^46^

### Host range testing

Spot testing was carried out; 5 μL of phage stock serial dilutions was plated on to bacterial lawn, in 0.4% overlay agar. Zones of bacterial lawn clearing, indicating cell lysis, were recorded as follows: (1) visible plaques, (2) complete bacterial lawn clearing, (3) turbid bacterial lawn, or (4) no effect. The presence or absence of halos; reduced turbidity of the bacterial lawn surrounding the plaques or clearing were also recorded.

### Plaque formation and morphology

Phages were plated by using the overlay agar plaque assay method, as described earlier, on their isolation host. Plates were incubated overnight at 37°C to allow plaques to form. Plaque morphology was noted (halos/no halo), and photographs were taken.

### Transmission electron microscopy

Pure phage stocks were imaged by transmission electron microscopy (TEM) on glow-discharged (1 min under vacuum) formvar/carbon-coated copper grids (Agar Scientific Ltd, United Kingdom). Five microliters of phage stock was applied to a grid and incubated for 1.5 min at room temperature. The grid was blotted to remove excess liquid. A drop of 2% uranyl acetate stain was applied and incubated for 1 min, before blotting off; staining was repeated four times; and finally the grid was air dried. Stained phage grids were imaged on a JEOL 2100Plus TEM. The morphology of the phage particles was visualized in ImageJ; 30 capsids and tails for each phage isolate were measured by using the measure function.

### Lysis period

*Klebsiella* cultures in the exponential growth phase were adjusted to OD_600nm_ of 0.2, using a spectrophotometer and phage lysates were diluted 1:4. The OD_600nm_ was measured every 5 min for 16 h. Growth was compared with a positive control culture without the addition of phages. The lysis period was calculated by measuring the time from phage addition to a drop in culture OD_600nm_, relative to the positive control, indicating bacterial cell lysis.

### Virulence

Two metrics the virulence index (VP) and MV50 were calculated based on the protocol described by Storms et al.^47^ Briefly, bacterial cultures were grown to the exponential phase and adjusted (see Lysis Period section) to an optical density equivalent to 1 × 10^8^ cfu/mL.

In a 96-well plate, phages were serially diluted from 1 × 10^8^ to 10 pfu/mL in 100 μL volumes. A bacterial culture was then added in equal volume (100 μL) to the phage dilution, resulting in multiplicity of infections (MOIs) from 1 to 10^−7^. The optical density of the 96-well plate was read at 600 nm at 5 min intervals for 18 h.

The area under the curve was calculated for the bacterial-only control and at each phage MOI, from initial infection until the exponential growth stage. The VP at each MOI was calculated from this by following the method described^47^ using RStudio (version 1.1.463). VP is a quantified measure of the virulence of a phage against a bacterial host on a scale of 0–1 (0 = no reduction in bacterial growth to 1, instantaneous complete killing); the MV50 calculates the theoretical MOI at which a phage achieves a VP of 0.5 (half the theoretical maximum virulence).

### Data visualization

Resulting graphs were visualized in R (v3.6.1) implemented through RStudio (v1.1.456)^48^ by using the ggplot2 (v3.3.2) package,^49^ with a custom color-blind palette generated from ColorBrewer.

## Results

### *Klebsiella* capsule and LPS antigen locus types

Kaptive determined that the 24 *Klebsiella* strains belonged to 18 different capsule (K) and LPS antigen (O) locus types (Table 1). Three *K. pneumoniae* strains were identified as capsule type KL2; two *Klebsiella* sp. were each typed as O1v1, OL104, and KL14. All other types were unique. *K. aerogenes* 30053 could not be typed.

### Sequence similarity to known phages

**FIG. 1.**
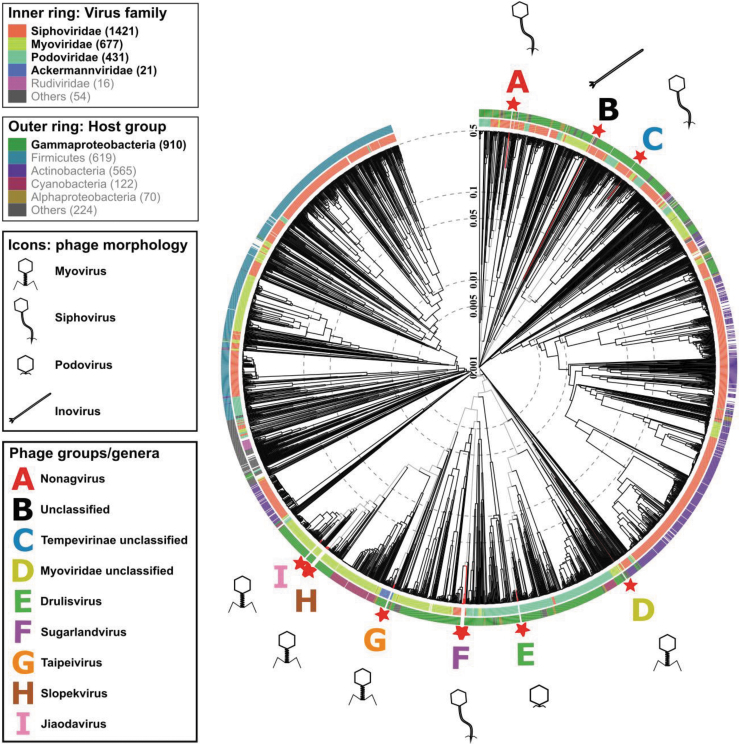
Protein-level phylogenetic tree, generated by VIPtree. *Klebsiella* phage isolates (★). A–I denote phage groups with the following genera: A (red) *Nonagvirus*; B (black) unclassified family/genus; C (blue) *Tempevirinae* unclassified; D (lime) *Myoviridae* unclassified; E (green) *Drulisvirus*; F (purple) *Sugarlandvirus*; G (orange) *Taipeivirus*; H (brown) *Slopekvirus*; and I (pink) *Jiaodavirus*. Icons indicate phage morphology.

**FIG. 2.**
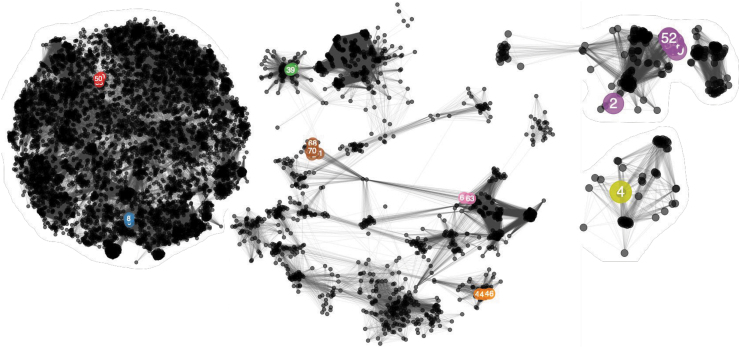
Network analysis of phage-encoded proteins calculated with vConTACT2. Colored, numbered nodes represent our Klebsiella phage isolates, colored according to the phage group and subsequent genera to which each phage belongs. Numbers within nodes indicate the lab identification numbers (Table 2). Our Klebsiella phage isolates are colored according to the genera to which the phage belongs: red, *Nonagvirus*; gray, unclassified; blue, *Tempevirinae* unclassified; lime, *Myoviridae* unclassified; green, *Drulisvirus*; purple, *Sugarlandvirus*; orange, *Taipeivirus*; brown, *Slopekvirus*; and Pink, *Jiaodavirus*. The numbers are the lab identification numbers (Table 2). Smaller, black nodes represent previously sequenced phages as references. Edges between nodes represent shared proteins, such that many connecting edges imply greater pairwise shared protein content. Phage nodes are clustered based on shared proteins, with a spring-embedded (force directed) layout visualization created in Cytoscape.

The 30 *Klebsiella* phages were purified by multiple rounds of plating, and genome sequencing showed a genome size range from 16,548 to 268,500 bp. The *Klebsiella* phage genomes represented nine diverse, distinct genera, as determined by VIPtree (Fig. 1) and vConTACT2 (Fig. 2). Genome similarities between our phage isolates and known phages are given in Table 3.

**Table 3.** Phage Taxonomy and Similarity to Closest Sequenced Phage

Phage name	Phage group	Taxonomy			Comparison phage		
		Family	Subfamily	Genus	Phage name	Accession	ANI
vB_KppS-Eggy	A	*Siphoviridae*		*Nonagvirus*	Enterobacteria phage JenP2	KP719132	65.86
vB_KppS-Pokey	A	*Siphoviridae*		*Nonagvirus*	Enterobacteria phage JenP2	KP719132	66.69
vB_KppS-Raw	A	*Siphoviridae*		*Nonagvirus*	Enterobacteria phage JenP2	KP719132	66.77
vB_KppS-Ant	B	*Siphoviridae*		unclassified	Caudovirales_phage_clone_3F_1	MF417951	90.14
vB_KaS-Ahsoka	C	*Drexlerviridae*	*Tunavirinae*	unclassified	Escherichia phage Henu7	MN019128	92.09
vB_KaS-Gatomon	C	*Drexlerviridae*	*Tunavirinae*	unclassified	Escherichia phage Henu7	MN019128	93.55
vB_KppS-Samwise	C	*Drexlerviridae*	*Tunavirinae*	unclassified	Escherichia phage Henu7	MN019128	93.55
vB_KvM-Eowyn	D	*Myoviridae*	*Tempevirinae*	unclassified	Serratia_phage_KpHz_2	KF806589	95.02
vB_KpP-Screen	E	*Autographiviridae*	*Slopekvirinae*	*Drulisvirus*	Klebsiella phage vB_KpnP_SU552A	KP708986	86.58
vB_KpP-Yoda	E	*Autographiviridae*	*Slopekvirinae*	*Drulisvirus*	Klebsiella phage vB_KpnP_SU552A	KP708986	87.03
vB_KqP-Goliath	E	*Autographiviridae*	*Slopekvirinae*	*Drulisvirus*	Klebsiella phage vB_KpnP_SU552A	KP708986	85.82
vB_KaS-Benoit	F	*Demerecviridae*		*Sugarlandvirus*	vB_Kpn_IME260	NC_041899	94.29
vB_KaS-Veronica	F	*Demerecviridae*		*Sugarlandvirus*	★Klebsiella phage Sugarland	NC_042093	93.46
vB_KppS-Anoxic	F	*Demerecviridae*		*Sugarlandvirus*	vB_Kpn_IME260	NC_041899	96.11
vB_KppS-Jiji	F	*Demerecviridae*		*Sugarlandvirus*	★Klebsiella phage Sugarland	NC_042093	93.26
vB_KppS-Ponyo	F	*Demerecviridae*		*Sugarlandvirus*	★Klebsiella phage Sugarland	NC_042093	94.15
vB_KppS-Storm	F	*Demerecviridae*		*Sugarlandvirus*	★Klebsiella phage Sugarland	NC_042093	95.02
vB_KppS-Totoro	F	*Demerecviridae*		*Sugarlandvirus*	vB_Kpn_IME260	NC_041899	94.29
vB_KqM-Bilbo	G	*Ackermannviridae*		*Taipeivirus*	★Klebsiella virus 0507KN21	NC_022343	97.22
vB_KqM-LilBean	G	*Ackermannviridae*		*Taipeivirus*	★Klebsiella virus 0507KN21	NC_022343	97.83
vB_KqM-Westerburg	G	*Ackermannviridae*		*Taipeivirus*	★Klebsiella virus 0507KN21	NC_022343	96.93
vB_KoM-Liquor	H	*Myoviridae*	*Tevenvirinae*	*Slopekvirus*	★Klebsiella phage KP15	GU295964	97.83
vB_KoM-MeTiny	H	*Myoviridae*	*Tevenvirinae*	*Slopekvirus*	★Klebsiella phage KP15	GU295964	97.92
vB_KoM-Pickle	H	*Myoviridae*	*Tevenvirinae*	*Slopekvirus*	★Klebsiella phage KP15	GU295964	97.93
vB_KpM-KalD	H	*Myoviridae*	*Tevenvirinae*	*Slopekvirus*	★Klebsiella phage KP15	GU295964	97.42
vB_KpM-Mild	H	*Myoviridae*	*Tevenvirinae*	*Slopekvirus*	★Klebsiella phage KP15	GU295964	97.65
vB_KpM-Milk	H	*Myoviridae*	*Tevenvirinae*	*Slopekvirus*	★Klebsiella phage KP15	GU295964	98.38
vB_KpM-SoFaint	H	*Myoviridae*	*Tevenvirinae*	*Slopekvirus*	★Klebsiella phage KP15	GU295964	97.72
vB_KoM-Flushed	I	*Myoviridae*	*Tevenvirinae*	*Jiaodavirus*	★Klebsiella phage JD18	KT239446	96.19
vB_KpM-Wobble	I	*Myoviridae*	*Tevenvirinae*	*Jiaodavirus*	★Klebsiella phage JD18	KT239446	97.01

Family, Subfamily, and Genus are assigned based on the clustering patterns observed in vConTACT2 analysis (Fig. 2), and conserved branching patterns observed in the marker gene phylogenetic trees (Supplementary Figs. S10–S18). ANI calculated with orthoANI. Details for “comparison phage” relate to details of previously sequenced phages available in public databases used for ANI analysis, ★ in front on the comparison phage name indicates it is the type species.

ANI, average nucleotide identity.

Phage isolates were grouped at genus-level into groups A–I, referred to, respectively, by their genera or closest identifiable taxonomy level: *Nonagvirus*, unclassified family/genus, *Tempevirinae* unclassified, *Myoviridae* unclassified, *Drulisvirus*, *Sugarlandvirus*, *Taipeivirus*, *Slopekvirus*, and *Jiaodavirus*. The phage isolates of groups B–D displayed lower sequence similarities to previously identified phages, hence they were not classified into known genera. The sequence data were deposited in the ENA; accession numbers are given in Table 2.

Alignments constructed in VIPtree^33^ showed that the phage groups with the highest levels of amino acid identity and gene synteny to known phages were F, G, H, and I (*Drulisvirus*, *Sugarlandvirus*, *Taipeivirus*, and *Jiaodavirus* genera, respectively; Supplementary Figs. S6–S9). Phage isolates in group F, *Sugarlandvirus*, showed the greatest similarity to previously described phages (Supplementary Fig. S6). All *Sugarlandvirus* isolates were grouped with previously described vB_Kpn_IME260 and *Klebsiella* phage Sugarland, except vB_KaS-Veronica, which represents a new species based on ANI. The *Sugarlandvirus* isolates did exhibit variation in their tail fiber genes (Supplementary Fig. S7; at ∼75 kb). In contrast, phage isolates in group A (*Nonagvirus*) were more similar to each other than previously known phages (Supplementary Fig. S2).

There was low sequence identity between our isolates and previously sequenced phages for groups B–D (Supplementary Figs. S3–S5); as a result, the isolates in these groups have unresolved taxonomies. Interestingly, analysis of the phage with the smallest genome, vB_KppS-Ant of group B, revealed >99% nucleotide identity to region of the *K. pneumoniae* 30104 genome (data not shown).

For further similarity analysis between our isolates and known phages, protein phylogenetic trees were drawn for marker genes (DNA polymerase, major capsid protein and terminase large subunit; Supplementary Figs. S10–S18). Conserved branching patterns, indicating close evolutionary history, were observed for groups A and E–I, confirming that they belong to known genera (Supplementary Figs. S10 and S14–S18), whereas groups B–D (Supplementary Figs. S11–S13) do not.

Given more distant relationships between the marker protein sequences, it is proposed that group C represents a novel genus of the subfamily *Tempevirinae* (Supplementary Fig. S12) and group D represents a novel genus of the *Myoviridae* family (Supplementary Fig. S13), whereas the marker genes of the phage isolate of group B has an even more distant relationship with phages of both *Siphoviridae* and *Myoviridae* families (Supplementary Fig. S11). Given the inovirus morphology observed for group B (Fig. 5, black box), classification at the family level also remains unresolved.

### Host range testing

**FIG. 3.**
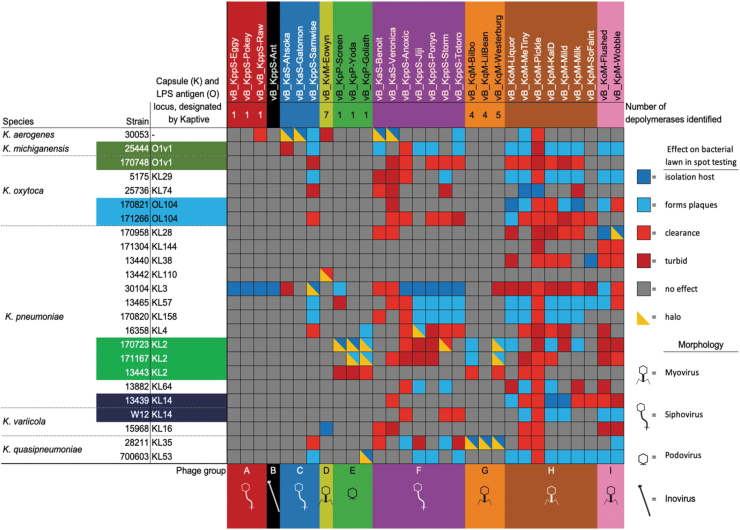
Phage host range matrix. Host range was determined by spot testing on LB agar overlay plates against the *Klebsiella* spp. in our panel. The *Klebsiella* capsule and LPS antigen locus have been designated by Kaptive. The phage names labels are colored according to the phage group and subsequent genera to which each phage belongs: A (red) *Nonagvirus*; B (black) unclassified family/genus; C (blue) *Tempevirinae* unclassified; D (lime) *Myoviridae* unclassified; E (green) *Drulisvirus*; F (purple) *Sugarlandvirus*; G (orange) *Taipeivirus*; H (brown) *Slopekvirus*; and I (pink) *Jiaodavirus*. The number of putative depolymerases identified by BLAST/HMMER analysis is given under the phage name. In the matrix, dark blue indicates the host of isolation, in which plaques were produced; light blue indicates the non-host strains where plaques were produced in a bacterial lawn; red indicates that spot testing caused the bacterial lawn to clear, but no plaques were visible; dark red indicates that some reduction in the turbidity of the bacterial lawn was observed, but no plaques; gray indicates no observed effect; and yellow triangle overlays indicate that a halo of reduced turbidity of the bacterial lawn was observed surrounding the plaques or clearing. LB, lysogeny broth; LPS, lipopolysaccheride.

Most phages had a host range that extended past their isolation host and was not explained by depolymerase activity. The number of strains infected by each phage is displayed in Figure 3.

The infection range of the two putatively temperate phage genera was limited to their isolation host; *Nonagvirus* (except vB_KppS-Raw, which infected one additional strain) and unclassified family/genera (B).

The lytic phage genera *Myoviridae* unclassified, *Drulisvirus* and *Taipeivirus* cleared a bacterial lawn in three to seven *Klebsiella* strains and formed plaques in one to seven of those strains. *Sugarlandvirus* phages showed a clearance of 16 and produced plaques in 10 *Klebsiella* strains; *Tempevirinae* phages were highly variable, with vB_KaS-Gatomon and vB_KaS-Ahsoka forming plaques on only their isolation host; and vB_Kpp-Samwise formed plaques in seven strains and cleared a further five strains.

The broadest range were lytic phages belonging to the subfamily *Tevenvirinae*; *Slopekvirus* and *Jiaodavirus*, which demonstrated clearance in 23 and 17 strains, respectively, and produced plaques on 13 and 9 strains, respectively. Within these genera, the host range varied between individual phages; from *Slopekvirus*, phage vB_KoM-Pickle only formed plaques on its isolation host, yet showed lawn clearance in 22 out of 23 *Klebsiella* spp. (Fig. 3).

### Phage annotation

Genome annotation is notoriously difficult with phages, given the extremes of sequence variation evident in all phage proteins,^50,51^ PROKKA was used and identified key phage genes, for example, portal proteins, capsid genes, tail proteins, and components of the DNA replication. In addition, PhoH was a common feature in 18 out of 30 phages sequenced, including phages from *Myoviridae* unclassified, *Sugarlandvirus*, *Taipeivirus*, and *Slopekvirus*, respectively. Holin and lysin pairs were identified in groups *Nonagvirus*, *Tempevirinae* unclassified, and *Taipeivirus*, whereas endolysin and Rz1 spanin complex genes were identified in *Drulisvirus* and *Jiaodavirus*.

Most of our isolated phages encode at least one gene annotated as a putative “tail-fibre” or “tail-spike” protein (Supplementary Table S2). Structural predictions suggested that these proteins adopt beta-helical structures, a common protein architecture of tail-spike proteins and capsule depolymerase enzymes, which are suggested to have evolved from these purely structural proteins,^52–54^ These proteins also contained predicted enzymatic domains, for example, Pectate_lyase_3 domain or Peptidase_S74 domain, which have been identified in other phage-encoded depolymerases.^55–58^ Several of the phage groups: B, C, F, H, and I, did not contain a predicted tail-fiber depolymerase protein.

**FIG. 4.**
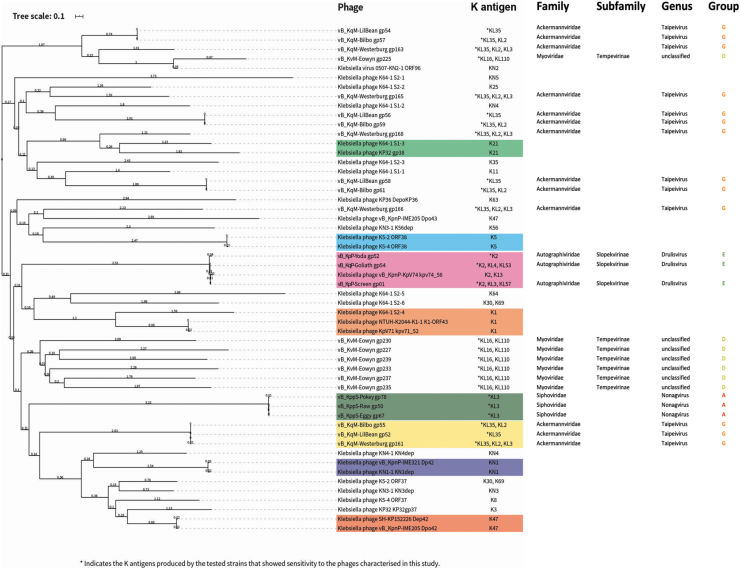
Phylogenetic tree of previously biochemically characterized depolymerase proteins that target *Klebsiella* spp. and putative depolymerase proteins in our phage isolates. Sequences were aligned with Muscle by using SeaView. Phylogenetic tree construction was performed with MegaX with 500 bootstrap calculations by using the LG model. Tree topology searches were performed by using a combination of NNI and NJ/BioNJ. The tree was subsequently visualized and annotated by using iTOL (v4). Depolymerases highlighted in color blocks have overlapping potential target K antigens.

For phages encoding a candidate depolymerase, protein sequence relationships were mapped on a tree (Fig. 4). Phages of the genera *Nonagvirus*, and *Drulisvirus* encode a similar predicted depolymerase protein. The putative depolymerases from *Drulisvirus* phages share high sequence conservation (∼95% identity, 100% query) to the experimentally characterized depolymerase Kpv74_56 from the closely related *Drulisvirus*, *K. pneumoniae* phage KpV74,^59^ and all these characterized phages infect K2 capsule-producing strains of *Klebsiella*; however, the *Nonagvirus* isolates in our study do not (Fig. 4).

Interestingly, the phages of two further genera; *Myoviridae* unclassified (vB_KqM-Eowyn) and *Taipeivirus* (vB_KqvM-LilBean, vB_KqvM-Bilbo and vB_KqvM-Westerburg) contain multiple putative depolymerase proteins (7, 4, 4, 5, respectively; Fig. 4). The putative depolymerase-like proteins of vB_KqM-Eowyn (*Myoviridae* unclassified) show low identity to previously characterized depolymerases (Fig. 4). From our host range assays, these phages showed activity toward strains that produced a small subset of K-antigens (vB_KqM-Eowyn—KL16, KL110; vB_KqvM-LilBean—KL35; vB_KqvM-Bilbo—KL35, K2 and vB_KqvM-Westerburg—KL35, KL2, KL3).

### Morphology of phages

**FIG. 5.**
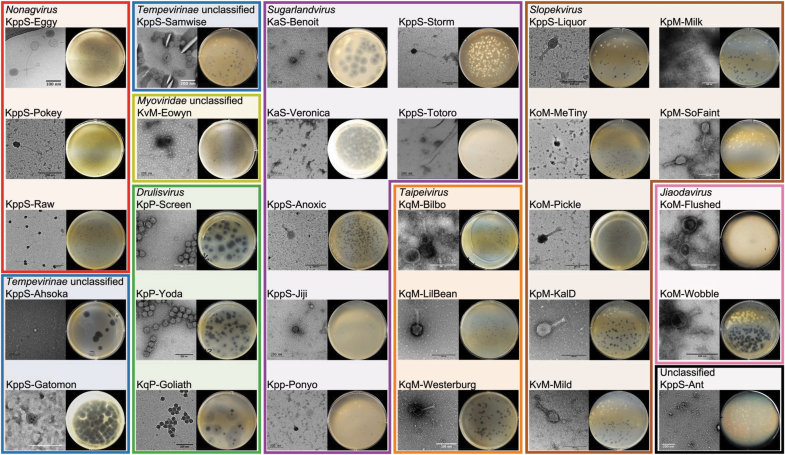
Phage morphology, TEM, and plaque morphology. Colored boxes are drawn according to the phage group and subsequent genera to which each phage belongs: A (red) *Nonagvirus*; B (black) unclassified family/genus; C (blue) *Tempevirinae* unclassified; D (lime) *Myoviridae* unclassified; E (green) *Drulisvirus*; F (purple) *Sugarlandvirus*; G (orange) *Taipeivirus*; H (brown) *Slopekvirus*; and I (pink) *Jiaodavirus*. The clearest TEM images were selected for each phage, scales vary for each image, and nm scale bars are included for each image. An image of the plaques produced by each phage is included after overnight incubation at 37°C on agar overlay plates. TEM, transmission electron microscopy.

Phage-induced plaques in the lawns of host bacteria varied in size, and in the presence/absence of halos surrounding the phage plaques. Representative images of phage plaques for each described genera are presented in Figure 5. A diffuse halo around the phage plaques was observed in 15 of the 30 phage isolates (Supplementary Table S3 and Fig. 5).

Representative TEM images of the described phage genera are provided in Figure 5. Average tail length and capsid widths are given in Supplementary Table S3 (based on 30 particles per phage), and a representative TEM image for each phage is given in Figure 5. Of the phages imaged, 13 are myovirus, 13 are siphovirus, 3 are podovirus, and 1 is inovirus.

The largest phage in this study was vB_KvM-Eowyn (*Myoviridae* unclassified), which had a capsid width of 140 nm and a tail length of 140 nm; this corresponded to the largest genome at 269 kbp. The smallest caudovirale phage was vB_KqP-Goliath, a podovirus (*Drulisvirus*), with a capsid width of 41 nm and tail length of 10 nm, had the second smallest genome at 44 kbp, after the filamentous prophage vB_KppS-Ant (unclassified family/genus). vB_KppS-Raw (*Nonagvirus*), a siphovirus, had a comparable capsid size of 46 nm, but a substantially longer tail (153 nm) and genome of 61 kbp. The smallest caudovirale phages (*Drulisvirus*) produced the largest plaques and halos, hence the name KqP-Goliath (Supplementary Table S1 and Fig. 5).

### Phage lysis period and virulence in host strains

**FIG. 6.**
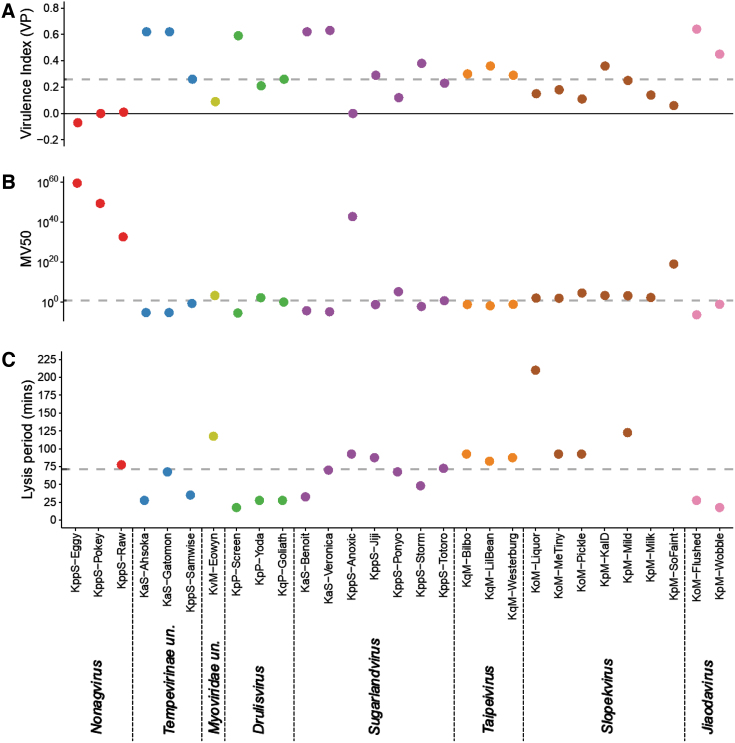
Lysis period and virulence indices are not correlated, but temperate phages are less virulent. **(A)** Displays the virulence index of the phages. Virulence index is a quantified measure of the phage, in their respective isolation host (Table 2), in LB with 5 mM each of CaCl_2_ and MgCl_2_, at 37°C. **(B)** displays the MV50, the MOI at which each phage achieves 50% of their maximal theoretical virulence. Both of these virulence measures are described in more detail by Storms.^47^ **(C)** Displays the lysis period of the phage. Where this is left blank, a lysis period could not be established, usually indicating temperate lifestyle. Individual points are colored based on phage group and the subsequent genera to which each phage belongs: A (red) *Nonagvirus*; C (blue) *Tempevirinae* unclassified; D (lime) *Myoviridae* unclassified; E (green) *Drulisvirus*; F (purple) *Sugarlandvirus*; G (orange) *Taipeivirus*; H (brown) *Slopekvirus*; and I (pink) *Jiaodavirus*. Dashed lines indicate the median values for each metric. MOI, multiplicity of infection.

The lysis period and virulence indices (VP and MV50) of each phage, in their relevant isolation host strain in LB at 37°C, are displayed in Figure 6. For six phages a lysis period was not achieved (vB_KppS-Eggy, vB_KppS-Pokey, vB_KppS-Ant, vB_KpM-Milk, vB_KpM-KalD, and vB_KpM-SoFaint), and the growth curve of the bacteria was dampened (except vB_KppS-Ant); however, the culture density did not crash compared with the positive control—indicative of temperate phages. These phages also demonstrated a below-average VP, close to 0 (with the exception of vB_KpM-KalD), indicating little difference in growth curves between the control and phage-infected cultures (Fig. 6).

Of the phages with a lysis period, the median time was ∼70 min, ranging from 15 to 210 min. There was no correlation between lysis period and the virulence measures (Fig. 6; statistical data not shown). The average VP was 0.33 (range 0.06–0.64) and MV50 was achieved with a projected MOI of 4.17 × 10^17^ (range 3.5 × 10^−7^ to 1 × 10^19^) for the lytic phage genera. The putatively temperate phages had lower virulence measures; average VP 0.06 (range −0.07 to 0.36) and MV50 returned a theoretical MOI of 6.00 × 10^58^ (range 0.012 to 3 × 10^59^).

*Tempevirinae* unclassified, *Drulisvirus*, *Sugarlandvirus*, and *Jiaodavirus* had the highest virulence (VP) (range 0.12–0.64, average 0.42) and below-average MV50 (range 3.5 × 10^−7^ to 1.30 × 10^5^, average 9.30 × 10^3^), indicating that they rapidly killed their hosts and needed a lower phage:host ratio to achieve this. It is, however, difficult to generalize for each genus, because the metrics varied drastically within genera. The virulence index results are specific to each phage and conditions assessed, and therefore they are not directly comparable between phages grown on different hosts.

## Discussion

*Klebsiella*-infecting phages, belonging to nine phylogenetically distinct lineages, were isolated from water samples sourced from different environments. The 30 phages were discovered by using a panel of *K. pneumoniae*, *K. oxytoca*, *K. quasipneumoniae*, *K. aerogenes*, and *K. variicola.* These *Klebsiella* spp. represent clinical and environmental strains that span 18 different capsule types.

This study discovered several phages and phage genera that have not previously been described. Phylogenetic analysis revealed that the filamentous phage vB_KppS-Ant did not significantly cluster with any known phages at the shared protein level, and therefore represents a novel genus. The genome reconstruction of this ssDNA phage is unexpected, however previously studies have demonstrated Illumina sequencing to be inefficient yet successful at sequencing ssDNA phages.^60^ The high sequence similarity of phage vB_KppS-Ant to a region of the *K. pneumoniae* 30104 genome (>99%) indicates that it is an induced prophage. By combining shared protein network analysis and marker gene phylogenetic tree analysis, we identified two further novel phage genera: *Tempevirinae* unclassified and *Myoviridae* unclassified.

### Factors determining host range

In general, comparative genomics revealed sequence conservation over a large portion of their genomes, with variability in only a few genes (Supplementary Figs. S4–S11), resulting in divergent characteristics in terms of host range and virulence (Figs. 3 and 6). Despite several of these phage species having been identified in previous studies, there are examples where these showed differences in host range. Thus, even within the small sample presented here, there is information to be gained about the factors determining host range.

It has been suggested that selection pressure imposed from the use of a host is sufficient to amplify nongenetic variants of a phage that can cross host -range,^47,61^ and it also remains possible that uncharacterized genes, encoding proteins of unknown function, could adapt a given phage to a distinct host.^62^ By way of example, *Sugarlandvirus* phages showed a high degree of similarity between genome sequences, with most variation concentrated in their tail fiber genes. Tail fibers mediate interaction with host cell receptors and are frequently rearranged in phages, allowing them to adhere to bacterial hosts.^63^ These tail fibers can include domains with enzymatic function, enabling degradation of host-specific features such as polysaccharide capsules.^64^

Three phages with >99% genome sequence similarity were isolated on two distinct hosts: *K. pneumoniae* 30104 (vB_KppS_Ponyo and vB_KppS_Totoro) or *K. aerogenes* 30053 (vB_KaS-Benoit). vB_KppS_Ponyo and vB_KaS-Benoit are 100% identical at the nucleotide level, and vB_KppS_Totoro has a single nucleotide polymorphism (SNP).

Host range analysis showed that the two phages propagated on *K. pneumoniae* 30104 had comparable host ranges, whereas the phage propagated on *K. aerogenes* 30053 had a slightly different host range (Fig. 3). This indicates that the propagation host, influenced host range, and that genome sequence alone cannot be used to infer host range.^65^ This is a vitally important consideration for phage therapy, and we cannot generalize phage behavior based on genome similarity, but we must also consider prevailing culture conditions.^66^

Although multiple features of strain-specific bacterial immunity can protect against phage replication, in *Klebsiella* the primary defense against both phages and antibiotics is a protective polysaccharide capsule.^67,68^ This capsule forms the outermost layer of the *Klebsiella* cell and acts as an important virulence factor.^69^ There are at least 77 different serologically defined *Klebsiella* capsule types.^70,71^ Whole-genome capsule typing^32^ showed that our *Klebsiella* panel encompassed 18 capsule types, including three KL2 strains, which are important in clinical infections and therefore of interest to develop effective therapies.^9^

*Klebsiella* phages have repeatedly shown to be specific to host capsule types,^72–74^ and this is often linked to phage sugar-degrading enzymes called depolymerases that target specific capsule types.^56,59,75^ Given the broad host ranges observed in our collection of phages (Fig. 3) and depolymerase-indicative halos^76^ in 83% of our phage isolates (Supplementary Table S3), we sought to identify depolymerase genes.

A surprisingly high number of putative depolymerase genes (7) were identified in vB_KvM-Eowyn (*Myoviridae* unclassified), which only produced plaques in its KL16 host strain, but showed potential depolymerase activity against a KL110-producing strain.

The *Taipeivirus* phages encoded four to six depolymerases, each of which also exceeded the number expected^53^; these phages produced plaques against the clinically relevant KL2 capsule type. These depolymerases have yet to be verified, and a wider panel of *Klebsiella* capsule types is necessary to confirm their activity. *Klebsiella* phages encoding up to 11 depolymerase genes have previously been characterized, but these infect a correspondingly wide range of *Klebsiella* capsule types,^77^ indicating a need to expand our *Klebsiella* panel.

*Nonagvirus* and *Drulisvirus* phages encoded one depolymerase gene each and demonstrated a small host range (Fig. 3). Characterization of these putative depolymerase genes will be important to further investigate the potential host range of these phages, beyond our current analysis. Genes encoding depolymerases were not identifiable in all of our halo-producing phages, including in some of the broadest ranging phages: *Sugarlandvirus*, *Slopekvirus*, and *Jiaodavirus*. The phage with the broadest host range, KoM-MeTiny (*Slopekvirus*), showed lawn clearance in 79% and produced plaques in 42% of the *Klebsiella* tested, which included nine different capsule types, but had no identifiable depolymerase genes. We suggest that this is either because they lack depolymerases or due to inadequate tools for depolymerase identification.^50,51^

There is limited sequence conservation between many of the putative tail-fiber/tail-spike depolymerase proteins from our phages and biochemically validated depolymerases, therefore further characterization will be critical to optimize phage cocktails for therapeutic use.

### Application to future phage-based therapy

None of our lytic phages was able to suppress *Klebsiella* growth for more than 12 h (Supplementary Fig. S1). In addition, selecting effective phages for the bacteria to be targeted can improve phage performance; phage vB_KoM_Liquor had an exceptionally long lysis period of 210 min, possibly due to poor propagation in the isolation host *K. oxytoca* 170821. Phage cocktails are used to improve the impact of phages on *Klebsiella* populations and are considered crucial for the efficacy of phage therapy.^78–80^ Phage cocktails benefit from complementarity and redundancy between the phages to overcome host-evolved phage resistance,^81^ which may account for the resurgences seen in our *Klebsiella* cultures.

After phage selection, the testing of phage combinations is essential to avoid adverse effects, which could result in bacterial stress responses or biofilm formation, as seen with sub-lethal antibiotic use.^82,83^ The phages described in this study have been supplied for use in compassionate phage therapy cocktails, requiring rigorous and lengthy testing to ensure safety and activity against the clinical *Klebsiella*.

For use in phage therapy, phages must not encode toxins or AMR genes.^78,80,84^ Fortunately, none of our *Klebsiella* phages contained either. Other factors, for example, the host infection dynamics and low virulence of the *Nonagvirus* indicate that these isolates may be temperate and/or may not be curative on infections. The temperate phage vB_KppS-Ant, as the only phage with an identifiable integrase for lysogeny, will be excluded for phage therapy purposes.^85^ *Jiaodavirus* phages encode a Hoc-like protein, which in phage T4 has been demonstrated to be highly immunogenic.^86^ It should be established as to whether these phages cause an immune response before using them for phage therapy.

The genomic information and experimental data presented here for *Tempevirinae* unclassified, *Sugarlandvirus*, *Taipeivirus*, and *Slopekvirus* indicate that they are lytic and, thus, suitable for preclinical evaluation. We suggest that the *Sugarlandvirus* and *Slopekvirus* isolates are the best candidates for future development in phage therapy, given their broad host range, high virulence, short latency period, and lack of potentially harmful genes. Taken together, our data suggest that to provide universal, effective phage therapy against *Klebsiella* infections, a phage cocktail comprising multiple diverse phages should be developed.

## Conclusions

A diverse range of *Klebsiella* phages were isolated. Despite some of our phage isolates being grouped into a single previously described phage species, within-species variation in both host range and virulence was observed. This demonstrates the necessity to microbiologically characterize phages for therapeutic use.

## Supplementary Material

Supplemental data

Supplemental data

Supplemental data

Supplemental data

Supplemental data

Supplemental data

Supplemental data

Supplemental data

Supplemental data

Supplemental data

Supplemental data

Supplemental data

Supplemental data

Supplemental data

Supplemental data

Supplemental data

Supplemental data

Supplemental data

Supplemental data

Supplemental data

Supplemental data
