# Supplementary material for: Isolation and Characterization of Klebsiella Phages for Phage Therapy
Source: Phage (New Rochelle). 2021 Mar 17;2(1):26–42. doi: 10.1089/phage.2020.0046 (PMC8006926; doi:10.1089/phage.2020.0046)
Supplement: Supplemental data [file Supp_Table2.docx]

Table S2. List of putative depolymerase tail-fibre proteins from novel phages characterised in this study. Each sequence was analysed by BLASTP, HMMer and HHpred using the default parameters. The top HHpred hits are described for each protein. These sequences in combination with the sequences of characterised depolymerases from phages described in Table S1 were used to construct the phylogenetic tree (Figure 4).

| **Phage** | **Family** | **Genus** | **Putative depolymerase** | **Protein Length (aa)** | **BLAST, HMMer, HHpred descriptions** | **HHpred Predicted boundaries (aa)** | **HHpred score**  **(E-value)** | **PDB code** |
| --- | --- | --- | --- | --- | --- | --- | --- | --- |
| vB_KvM-Eowyn | *Myoviridae* | unclassified | gp225 | 1183 | Bacteriophage CBA120 tailspike-protein4; hydrolase; Tail_spike_N  Tailspike domain protein gp42, Peptidase_S74, chaperone | 30-502  962-1117 | 0.0017  1.6e-11 | 5W6H  6EU4 |
|  |  |  | gp227 | 591 | Tail fiber protein, β-helical, pectate lyase  Particle associated glycoside hydrolase | 3-428  3-431 | 8.2e-21  2.0e-20 | 5W5P  6C72 |
|  |  |  | gp230 | 581 | K5 lyase  Tailspike-protein, beta-helix | 10-318  42-560 | 7.5e-14  7.1e-13 | 2X3H  4XOT |
|  |  |  | gp233 | 820 | Putative endo-N-neuraminidase; Outer surface protein A, hydrolase  K5 lyase | 1-420  57-548 | 5.6e-17  5.5e-15 | 5ZRU  2X3H |
|  |  |  | gp235 | 556 | Bacteriophage CBA120 tailspike-protein4; hydrolase  Tailspike-protein; parallel beta helix, putative endo-glycosidase | 12-533  12-400 | 1.2e-19  1.4e-15 | 5W6H  6NW9 |
|  |  |  | gp237 | 616 | Endo-xylogalacturonan hydrolase; Putative endo-N-acetylneuraminidase | 275-500 | 26 | 4CL2 |
|  |  |  | gp239 | 793 | Plasmin and fibronectin-binding protein A  Beta_Helix; Putative tail fiber; tailspike; hydrolase CBA120 | 83-582  40-521 | 7.8e-24  5.7e-21 | 4MR0  5W6S |
| vB_KppS-Raw | *Siphoviridae* | *Nonagvirus* | gp50 | 740 | Pectate_lyase_3 superfamily; Beta_helix, phage_tail_N;  Outer Surface Protein A; hydrolase  Plasmin and fibronectin-binding protein | 208-730  291-732 | 5.4e-21  6.1e-19 | 5ZRU  4MR0 |
| vB_KppS-Eggy | *Siphoviridae* | *Nonagvirus* | gp67 | 740 | Pectate_lyase_3 superfamily; Beta_helix, phage_tail_N;  Outer Surface Protein A; hydrolase  Plasmin and fibronectin-binding protein | 208-730  291-732 | 5.4e-21  6.1e-19 | 5ZRU  4MR0 |
| vB_KppS-Pokey | *Siphoviridae* | *Nonagvirus* | gp78 | 740 | Pectate_lyase_3 superfamily; Beta_helix, phage_tail_N;  Outer Surface Protein A; hydrolase  Plasmin and fibronectin-binding protein | 205-730  291-732 | 1.1e-21  5.5e-19 | 5ZRU  4MR0 |
| vB_KpvM-LilBean | *Ackermannviridae* | *Taipeivirus* | gp52 | 1039 | phiAB6 tailspike; beta helix; superhelical trimer  Tailspike protein; parallel beta helix, putative endo-glycosidase | 86-813  209-851 | 1.0e-21  6.4e-17 | 5JS4  6NW9 |
|  |  |  | gp54 | 728 | phiAB6 tailspike; beta helix; superhelical trimer  Tailspike protein; parallel beta helix, putative endo-glycosidase | 1-552  140-576 | 1.5e-19  1.2e-17 | 5JS4  6NW9 |
|  |  |  | gp56 | 735 | Tailspike protein  Beta-1,3-glucanase; cellulose, glucanase  Beta-1,3-glucanase; tandem beta-helix; glucosidase, hydrolase | 81-282  75-282 | 4.0e-16  9.0e-16 | 5M5Z  3EQN |
|  |  |  | gp58 | 663 | Tailspike protein; parallel beta helix, putative endo-glycosidase  Bacteriophage CBA120 tailspike-protein4; hydrolase | 1-490  18-480 | 8.2e-13  1.2e-11 | 6NW9  5W6H |
| vB_KpvM-Bilbo | *Ackermannviridae* | *Taipeivirus* | gp55 | 1039 | phiAB6 tailspike; beta helix; superhelical trimer  Tailspike protein; parallel beta helix, putative endo-glycosidase | 86-813  209-851 | 1.0e-21  6.4e-17 | 5JS4  6NW9 |
|  |  |  | gp57 | 728 | phiAB6 tailspike; beta helix; superhelical trimer  Tailspike protein; parallel beta helix, putative endo-glycosidase | 1-552  140-576 | 1.5e-19  1.2e-17 | 5JS4  6NW9 |
|  |  |  | gp59 | 747 | Particle-associated glycoside hydrolase; glycosidase; tailspike  Tail fiber protein; AM27, tailspike protein | 12-354  11-320 | 6.9e-14  1.7e-12 | 6C72  5W5P |
|  |  |  | gp61 | 663 | Tailspike protein; parallel beta helix, putative endo-glycosidase  Bacteriophage CBA120 tailspike-protein4; hydrolase | 1-490  18-480 | 8.2e-13  1.2e-11 | 6NW9  5W6H |
| vB_KpvM-Westerburg | *Ackermannviridae* | *Taipeivirus* | gp161 | 1039 | phiAB6 tailspike; beta helix; superhelical trimer  Tailspike protein; parallel beta helix, putative endo-glycosidase | 86-813  371-852 | 3.7e-20  1.8e-16 | 5JS4  6NW9 |
|  |  |  | gp163 | 735 | Bacteriophage CBA120 tailspike-protein4; hydrolase; Tail_spike_N  phiAB6 tailspike; beta helix; superhelical trimer  CBM22; Binding site, carbohydrates, enzyme stability | 77-735  1-492  577-735 | 1.7e-31  7.1e-15  0.00017 | 5W6H  5JS4  4XUO |
|  |  |  | gp165 | 284 | Tailspike protein; parallel beta helix, putative endo-glycosidase  Rhamngalacturonase A; hydrolase; parallel beta-helix glycosidase | 1-277  98-180 | 2.3e-9  5.5e-8 | 6NW9  1RMG |
|  |  |  | gp166 | 552 | Polygalacturonase, glycosylhydrolase  Endopolygalacturonase; beta helical structure; glycoside hydrolase | 6-280  7-280 | 3.1e-10  3.3e-10 | 1IB4  1K5C |
|  |  |  | gp168 | 675 | Depolymerase KP32gp38; Klebsiella pneumoniae capsule depolymerase  Tailspike protein; parallel beta helix, putative endo-glycosidase | 93-675  1-418 | 5.6e-107  5.6e-19 | 6TKU  6NW9 |
| vB_KpP-Yoda | *Autographiviridae* | *Drulisvirus* | gp52 | 464 | Bacteriophage CBA120 tailspike-protein4; hydrolase  Tailspike protein gp42  Endo-1,4-Beta-Xylanase Y; carbohydrate binding module | 4-464  2-267  307-464 | 1.0e-28  4.5e-11  0.0026 | 5W6H  6EU4  1DYO |
| vB_KpP-Goliath | *Autographiviridae* | *Drulisvirus* | gp54 | 577 | Bacteriophage CBA120 tailspike-protein4; hydrolase  Tailspike protein gp42  Endo-1,4-Beta-Xylanase Y; carbohydrate binding module | 1-576  8-407  420-576 | 1.2e-35  8.3e-25  0.0068 | 5W6H  6EU4  1DYO |
| vB_KpP-Screen | *Autographiviridae* | *Drulisvirus* | gp01 | 577 | Bacteriophage CBA120 tailspike-protein4; hydrolase  Tail fiber protein; AM27 Tailspike protein  Endo-1,4-Beta-Xylanase Y; carbohydrate binding module, Phage_Tail_middle | 3-576  8-355  420-577 | 1.1e-37  3.6e-24  0.003 | 5W6H  5W5P  1DYO |
