# Supplementary material for: Isolation and Characterization of Klebsiella Phages for Phage Therapy
Source: Phage (New Rochelle). 2021 Mar 17;2(1):26–42. doi: 10.1089/phage.2020.0046 (PMC8006926; doi:10.1089/phage.2020.0046)
Supplement: Supplemental data [file Supp_Fig9.docx]

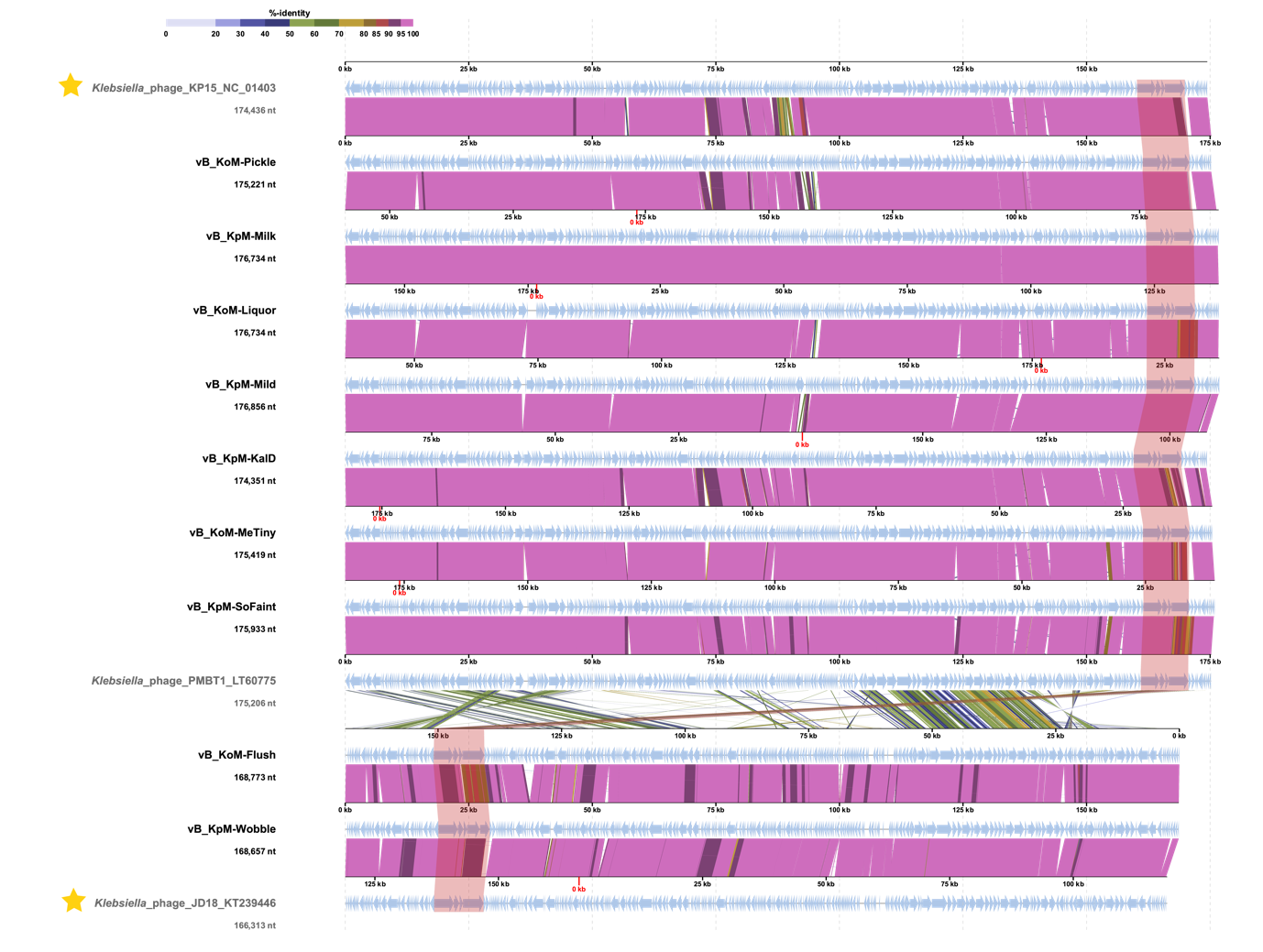


Figure S9. Combined group H (*Slopekvirus*) and group I (*Jiaodavirus*) amino acid alignment of our phage isolates and reference genomes (grey text), the type species are marked with yellow stars, identified in vConTACT2 analysis, drawn in VIPtree. Red shapes linking phages indicate the position of putative phage tail genes.
