# Supplementary material for: Isolation and Characterization of Klebsiella Phages for Phage Therapy
Source: Phage (New Rochelle). 2021 Mar 17;2(1):26–42. doi: 10.1089/phage.2020.0046 (PMC8006926; doi:10.1089/phage.2020.0046)
Supplement: Supplemental data [file Supp_Fig7.docx]

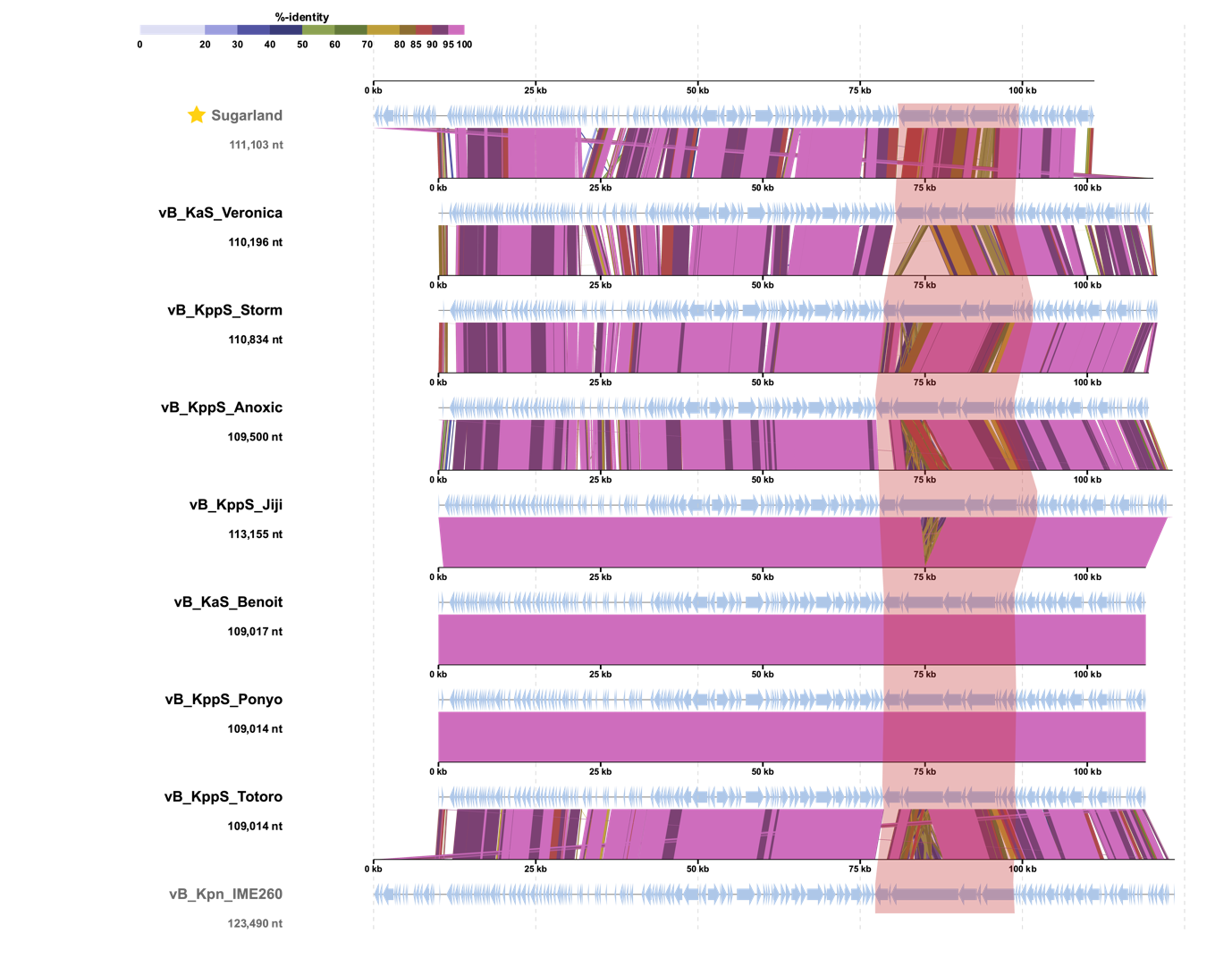


Figure S7. Group F (*Sugarlandvirus*) amino acid alignment of our phage isolates (black text) and reference genomes (grey text), the type species is marked with a yellow star, identified in vConTACT2 analysis, drawn in VIPtree. Red shapes linking phages indicate the position of putative phage tail genes.
