# Supplementary material for: Isolation and Characterization of Klebsiella Phages for Phage Therapy
Source: Phage (New Rochelle). 2021 Mar 17;2(1):26–42. doi: 10.1089/phage.2020.0046 (PMC8006926; doi:10.1089/phage.2020.0046)
Supplement: Supplemental data [file Supp_Fig3.docx]

*
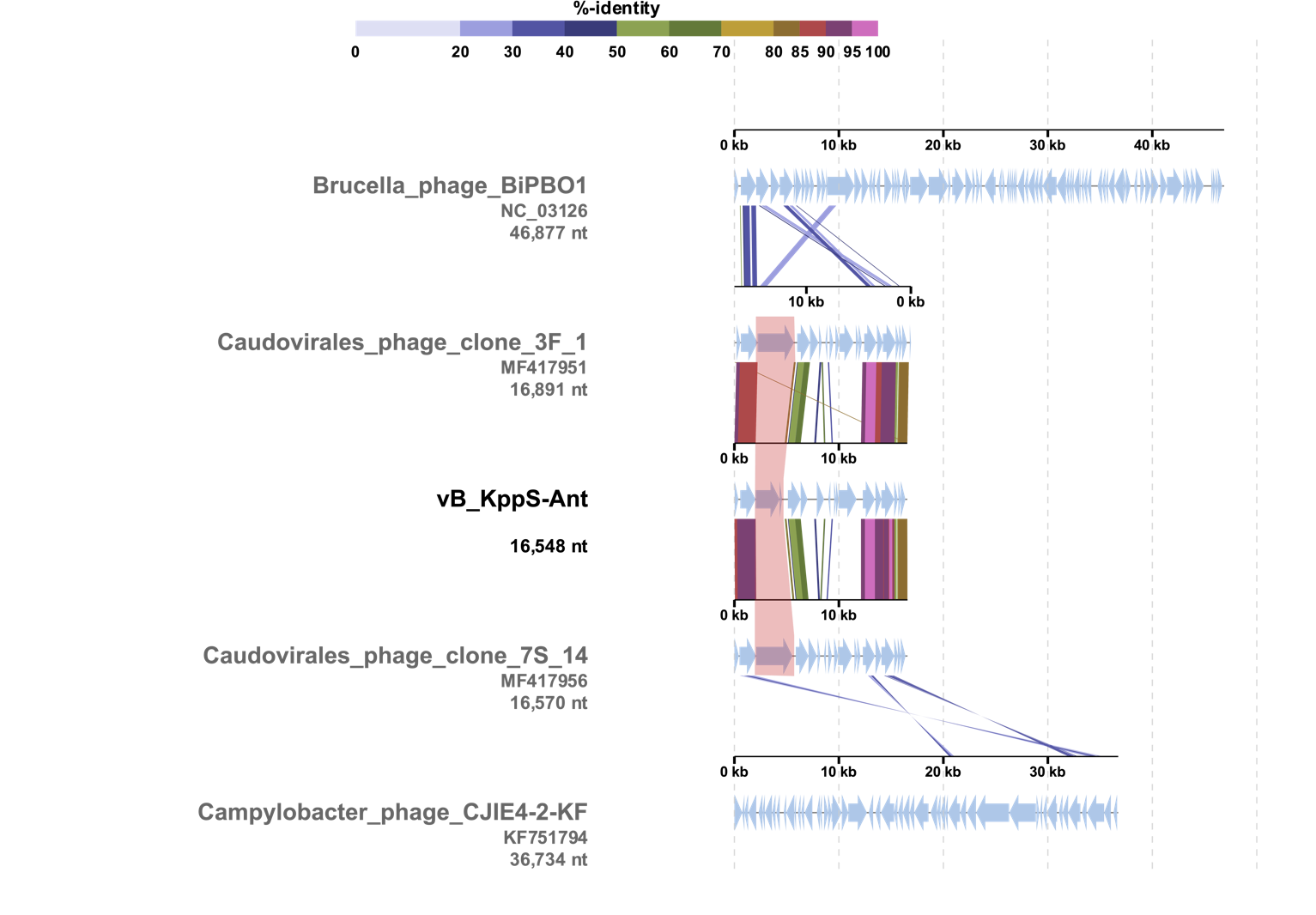
*

Figure S3. Group B (unclassified family/genus) amino acid alignment of our phage isolates (black text) and reference genomes (grey text) identified in vConTACT2 analysis, drawn in VIPtree. Red shapes linking phages indicate the position of putative phage tail genes.
