# Supplementary material for: Isolation and Characterization of Klebsiella Phages for Phage Therapy
Source: Phage (New Rochelle). 2021 Mar 17;2(1):26–42. doi: 10.1089/phage.2020.0046 (PMC8006926; doi:10.1089/phage.2020.0046)
Supplement: Supplemental data [file Supp_Table3.docx]

Table S3. Further phage characteristics. Phage names are coloured according to genera to which the phage belongs: A. (red) *Nonagvirus*; B. (black) unclassified family/genus; C. (blue) *Tempevirinae* unclassified; D. (lime) *Myoviridae* unclassified*;* E. (green) *Drulisvirus;* F. (purple) *Sugarlandvirus;* G. (orange) *Taipeivirus*; H. (brown) *Slopekvirus* and I. (pink) *Jiaodavirus*. Phages are ordered by genome size. Phage particle dimension, ability to form halos and presence of phoH-like gene in the genome sequence. Measurements; phage particle measurement calculated from TEM imaging and measured with imageJ, and genome size in nucleotide base pairs.

| Phage | Family | Subfamily | Genus | Tail length (nm) | Capsid width (nm) | Morphology | Genome size (bp) |
| --- | --- | --- | --- | --- | --- | --- | --- |
| vB_KppS-Ant | Unclassified |  |  | 403 | N/A | inoviridae | 16,548 |
| vB_KpP-Screen | *Autographiviridae* | *Slopekvirinae* | *Drulisvirus* | 14 | 53 | podoviridae | 43,749 |
| vB_KqP-Goliath | *Autographiviridae* | *Slopekvirinae* | *Drulisvirus* | 10 | 41 | podoviridae | 44,010 |
| vB_KpP-Yoda | *Autographiviridae* | *Slopekvirinae* | *Drulisvirus* | 13 | 51 | podoviridae | 44,122 |
| vB_KaS-Gatomon | *Drexlerviridae* | *Tempevirinae* | unclassified | 60 | 54 | siphoviridae | 49,702 |
| vB_KaS-Ahsoka | *Drexlerviridae* | *Tempevirinae* | unclassified | 200 | 60 | siphoviridae | 49,702 |
| vB_KppS-Samwise | *Drexlerviridae* | *Tempevirinae* | unclassified | 204 | 65 | siphoviridae | 49,891 |
| vB_KppS-Eggy | *Siphoviridae* |  | *Nonagvirus* | 141 | 55 | siphoviridae | 60,681 |
| vB_KppS-Raw | *Siphoviridae* |  | *Nonagvirus* | 153 | 46 | siphoviridae | 61,195 |
| vB_KppS-Pokey | *Siphoviridae* |  | *Nonagvirus* | 153 | 46 | siphoviridae | 61,218 |
| vB_KaS-Benoit | *Demerecviridae* |  | *Sugarlandvirus* | 180 | 55 | siphoviridae | 109,014 |
| vB_KppS-Totoro | *Demerecviridae* |  | *Sugarlandvirus* | 170 | 70 | siphoviridae | 109,014 |
| vB_KppS-Ponyo | *Demerecviridae* |  | *Sugarlandvirus* | 240 | 70 | siphoviridae | 109,014 |
| vB_KppS-Anoxic | *Demerecviridae* |  | *Sugarlandvirus* | 84 | 60 | siphoviridae | 109,500 |
| vB_KaS-Veronica | *Demerecviridae* |  | *Sugarlandvirus* | 160 | 60 | siphoviridae | 110,196 |
| vB_KppS-Storm | *Demerecviridae* |  | *Sugarlandvirus* | 203 | 69 | siphoviridae | 110,834 |
| vB_KppS-Jiji | *Demerecviridae* |  | *Sugarlandvirus* | 220 | 73 | siphoviridae | 113,155 |
| vB_KqM-Bilbo | *Ackermannviridae* |  | *Taipeivirus* | 118 | 78 | myoviridae | 158,858 |
| vB_KqM-LilBean | *Ackermannviridae* |  | *Taipeivirus* | 124 | 82 | myoviridae | 158,859 |
| vB_KqM-Westerburg | *Ackermannviridae* |  | *Taipeivirus* | 124 | 90 | myoviridae | 158,883 |
| vB_KpM-Wobble | *Myoviridae* | *Tevenvirinae* | *Jiaodavirus* | 83 | 83 | myoviridae | 168,530 |
| vB_KoM-Flushed | *Myoviridae* | *Tevenvirinae* | *Jiaodavirus* | 115 | 70 | myoviridae | 168,773 |
| vB_KpM-KalD | *Myoviridae* | *Tevenvirinae* | *Slopekvirus* | 124 | 93 | myoviridae | 174,351 |
| vB_KoM-Pickle | *Myoviridae* | *Tevenvirinae* | *Slopekvirus* | 121 | 65 | myoviridae | 175,221 |
| vB_KoM-MeTiny | *Myoviridae* | *Tevenvirinae* | *Slopekvirus* | 99 | 67 | myoviridae | 175,419 |
| vB_KpM-SoFaint | *Myoviridae* | *Tevenvirinae* | *Slopekvirus* | 120 | 78 | myoviridae | 175,933 |
| vB_KoM-Liquor | *Myoviridae* | *Tevenvirinae* | *Slopekvirus* | 117 | 67 | myoviridae | 176,734 |
| vB_KpM-Milk | *Myoviridae* | *Tevenvirinae* | *Slopekvirus* | 127 | 70 | myoviridae | 176,734 |
| vB_KpM-Mild | *Myoviridae* | *Tevenvirinae* | *Slopekvirus* | 107 | 82 | myoviridae | 176,856 |
| vB_KvM-Eowyn | *Myoviridae* |  | unclassified | 171 | 130 | myoviridae | 268,550 |
