# Supplementary material for: Isolation and Characterization of Klebsiella Phages for Phage Therapy
Source: Phage (New Rochelle). 2021 Mar 17;2(1):26–42. doi: 10.1089/phage.2020.0046 (PMC8006926; doi:10.1089/phage.2020.0046)
Supplement: Supplemental data [file Supp_Fig4.docx]

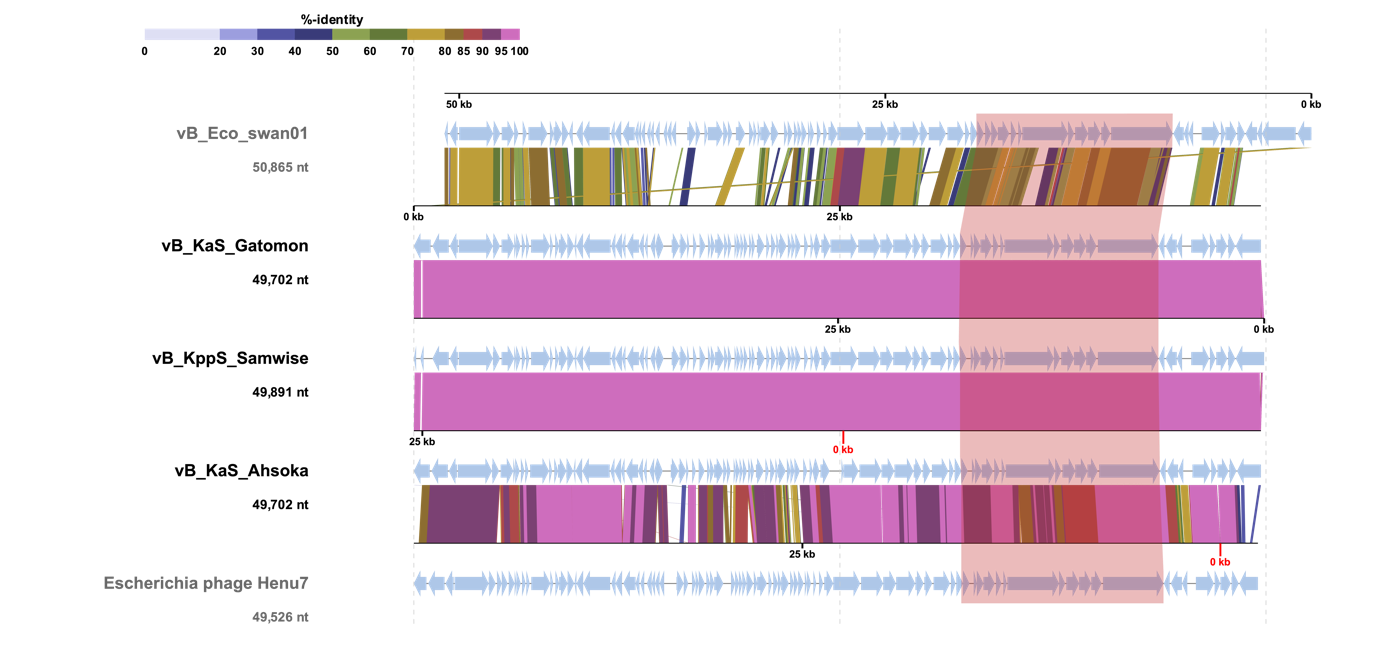


Figure S4. Group C (*Tempevirinae* unclassified) amino acid alignment of our phage isolates (black text) and reference genomes (grey text) identified in vConTACT2 analysis, drawn in VIPtree. Red shapes linking phages indicate the position of putative phage tail genes.
