# Supplementary material for: Isolation and Characterization of Klebsiella Phages for Phage Therapy
Source: Phage (New Rochelle). 2021 Mar 17;2(1):26–42. doi: 10.1089/phage.2020.0046 (PMC8006926; doi:10.1089/phage.2020.0046)
Supplement: Supplemental data [file Supp_Fig1.docx]

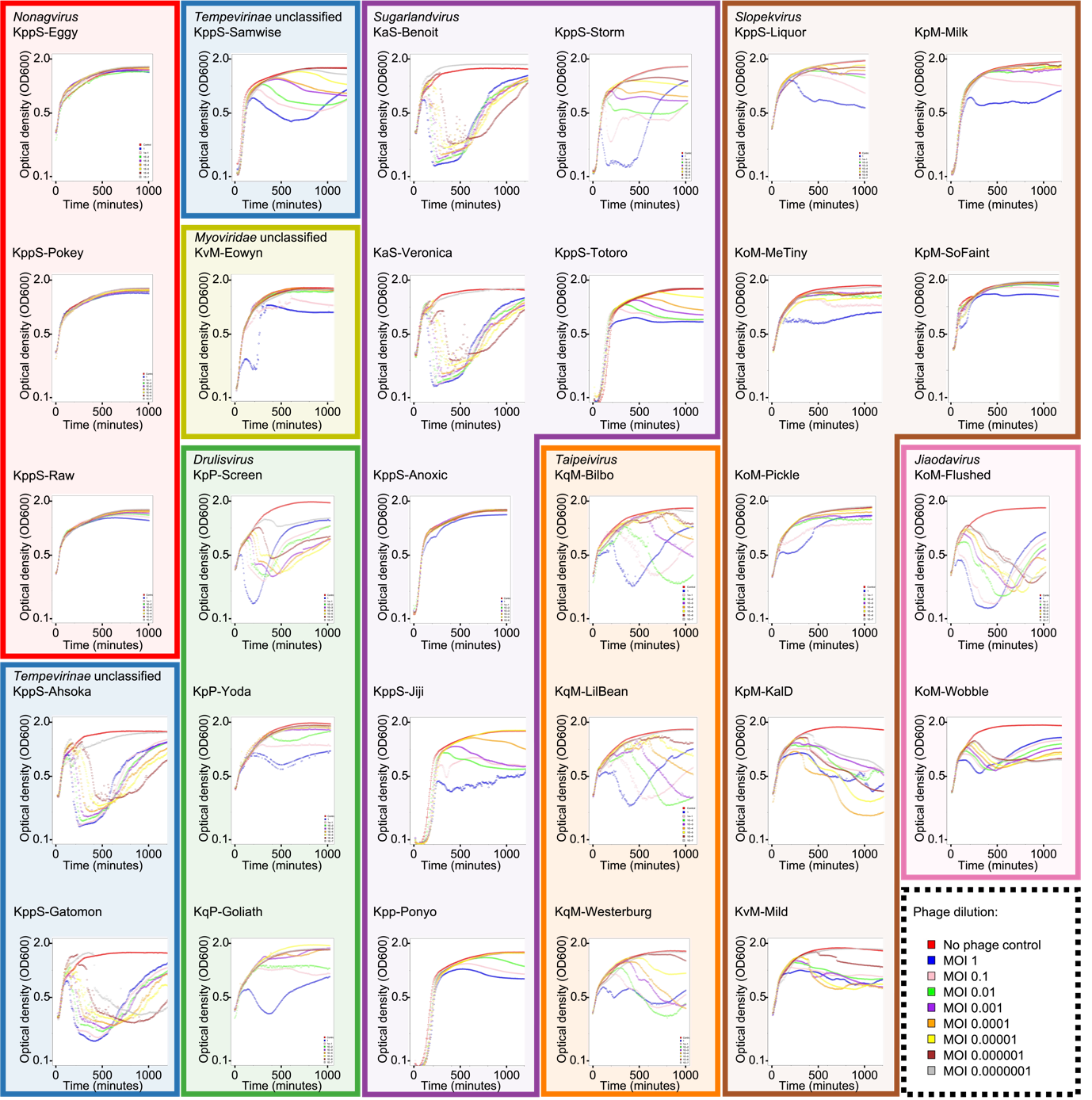


Figure S1. Impact of each phage isolate on the growth curves of its reciprocal isolation host adjusted to 1x10^8 cfu/mL, grown in LB at 37 °C. The key shows the phage dilutions added for every growth curve graph, an MOI of 1 is equal to 1x10^8 pfu/ml. Coloured boxes are drawn according to the phage group and subsequent genera to which each phage belongs: A. (red) *Nonagvirus*; B. (black) unclassified family/genus; C. (blue) *Tempevirinae* unclassified; D. (lime) *Myoviridae* unclassified*;* E. (green) *Drulisvirus;* F. (purple) *Sugarlandvirus;* G. (orange) *Taipeivirus*; H. (brown) *Slopekvirus* and I. (pink) *Jiaodavirus*.
