# Supplementary material for: Isolation and Characterization of Klebsiella Phages for Phage Therapy
Source: Phage (New Rochelle). 2021 Mar 17;2(1):26–42. doi: 10.1089/phage.2020.0046 (PMC8006926; doi:10.1089/phage.2020.0046)
Supplement: Supplemental data [file Supp_Fig17.docx]

Figure S17. Phylogenetic trees of DNA polymerase, major capsid protein and terminase large subunit of phage Group H (*Slopekvirus*). Drawn with RaxML using the GAMMA model of heterogeneity and the maximum-likelihood method based on the JTT substitution matrix. Branch labels indicate bootstrap values (100 replicates), representing the percentage of trees with the associated taxa clustering, are shown next to the branches. The scale bar represents the average number of substitutions per amino acid. Trees contain phage isolates from group H (brown) and reference phages (black) identified by vConTACT2 and closest BLASTP hits. Coloured bar indicates known genera of reference phages. Yellow stars indicate type species for reference phage genera.
